# Afatinib Targeted Therapy Affects the Immune Function and Serum Levels of EGFR and Gastrin-Releasing Peptide Precursor (pro-GRP) in Patients with Non-Small-Cell Lung Cancer (NSCLC)

**DOI:** 10.1155/2022/2869531

**Published:** 2022-09-14

**Authors:** Wei Cao, Jun Ma, Xuan Jiang, Guangyi Gao

**Affiliations:** ^1^Department of Pulmonology, Hongze District, Huai'an City Hospital of Traditional Chinese Medicine, Huai'an, 223100 Jiangsu, China; ^2^Department of Oncology, The Affiliated Huai'an Hospital of Xuzhou Medical University and The Second People's Hospital of Huai'an, No.60, Huaihai Road (S.), Huai'an, 223002, Jiangsu, China; ^3^Department of Oncology, Huai'an Second People's Hospital, Affiliated to Xuzhou Medical University, Huaian, 223000 Jiangsu, China

## Abstract

**Objective:**

This study is aimed at investigating the clinical intervention effect of afatinib targeted therapy in patients with non-small-cell lung cancer.

**Methods:**

The research object was a retrospective analysis of 86 patients with non-small-cell lung cancer who were admitted to our hospital from 1^st^ January 2019 to 31^st^ December 2021. The patients were divided into two groups. The patients in the two groups received conventional chemotherapy intervention, and the patients in group B received afatinib targeted therapy intervention on the basis of the treatment in group A. The clinical intervention effect, immune function, serum EGFR level, serum pro-GRP level, and incidence of adverse reactions were compared between the two groups of patients.

**Results:**

After afatinib targeted therapy intervention, the total intervention effective rate of patients in treatment group B was significantly higher than that in patients in treatment group A. Compared with the treatment group A, the CD3+, CD4+, CD8+, and CD4+/CD8+ of the treatment group were significantly upregulated. After the intervention, the serum EGFR levels of patients in treatment groups A and B were significantly decreased, and the serum EGFR levels in patients in treatment group B were significantly lower than those in patients in treatment group A. The serum pro-GRP level in group B patients was significantly decreased. The overall incidence of adverse reactions in treatment group B was significantly lower than that in treatment group A.

**Conclusion:**

Afatinib targeted therapy has a significant clinical intervention effect on patients with non-small-cell lung cancer, which not only helps to improve the immune function of patients but also effectively improves the serum EGFR and pro-GRP levels of patients.

## 1. Introduction

Non-small-cell lung cancer (NSCLC) is one of the most common malignant tumors in the clinic, and most patients have already entered an advanced stage when diagnosed, missing the best time for treatment [1]. At present, molecular targeted therapy is a new method of drug treatment for non-small-cell lung cancer, among which afatinib targeted therapy has a significant clinical effect [2, 3]. Some research literatures have found that afatinib targeted therapy can treat epidermal growth factor receptor kinase as a therapeutic target and then effectively inhibit the patient's cancer cells, thereby achieving an anticancer effect [4, 5]. In this study, a total of 86 patients with non-small-cell lung cancer who were treated in our hospital were selected as the main observation objects of this research analysis, and the clinical intervention effect, immune function, serum EGFR level, and serum progesterone after targeted therapy with afatinib were further observed, aiming to provide a basis for clinical intervention in patients with non-small-cell lung cancer.

## 2. Materials and Methods

### 2.1. Patient Information

A total of 86 patients with non-small-cell lung cancer who met the inclusion and exclusion criteria finally collected by our hospital from 1^st^ January 2019 to 31^st^ December 2021 were included in the study. Patients in group A received routine chemotherapy intervention, while patients in group B received afatinib targeted therapy intervention based on the treatment of group A. The study protocol was approved by the ethics committee of Huaian City Hospital of traditional Chinese medicine. All patients signed the consent form. Inclusion criteria [6]: (1) all included patients met the corresponding inclusion criteria; (2) all included patients had a survival period of ≥12 months; (3) all included patients were 47-82 years old. Exclusion criteria [7]: (1) the included patients are pregnant women; (2) the included patients have severe mental illness and cannot complete the study independently.

### 2.2. Treatment

Treatment group A (conventional chemotherapy intervention): on the 1st and 8th days, patients were given 100 mg/m^2^ gemcitabine hydrochloride mixed by intravenous drip (Qilu Pharmaceutical Co., Ltd., H20113285) and 250 ml sodium chloride injection (Harbin triple drug Industrial Co., Ltd., approved by Chinese medicine H20184091), 30 mg/m^2^ cisplatin (Qilu Pharmaceutical Co., Ltd., approved by Chinese medicine, H37021362) was intravenously infused on the second, third, and fourth days, once a day, for 3 months. Treatment group B (targeted therapy intervention with afatinib based on the treatment of group A): patients were given warm water orally with afatinib (Boehringer Ingelheim Pharma GmbH & Co., Germany, National Medicine Zhunzi J20170028), 3 times a day, continuous treatment 3 months.

### 2.3. Evaluation of Clinical Intervention Effect

Basic improvement: the malignant tumor lesions of the enrolled patients were basically eliminated; partial improvement: the malignant tumor lesions of the enrolled patients shrunk by more than half; stable disease: more than half of the malignant tumor lesions of the enrolled patients and the expansion of the lesions is less than 1/3; stable disease: the enrolled patients' new lesions of malignant tumors. Total effective rate of intervention = (basic improvement + partial improvement)/total number of cases × 100% [8, 9].

### 2.4. Immune Function Assessment

Before and after the intervention, 10 ml of venous blood was drawn from all the included patients on an empty stomach in the morning, centrifuged for 10 minutes, and then, the CD3+, CD4+, and CD4+/CD8+ immune indexes of the included patients were detected and analyzed by enzyme-linked immunosorbent assay. The kit was purchased in Beijing Biolab Technology Co., Ltd., the intrabatch variation is kept below 10%, and the interbatch variation is kept below 15%.

### 2.5. Detection of Serum EGFR and pro-GRP Levels

Before and after the intervention, 10 ml of venous blood was drawn from all the included patients on an empty stomach in the morning, centrifuged for 10 min, and then, the serum epidermal growth factor receptor (EGFR) and serum gastrin levels of the included patients were measured by enzyme-linked immunosorbent assay method. The level of released peptide precursor (pro-GRP) was detected and analyzed. The kits were purchased from Shanghai Jingkang Bioengineering Co., Ltd. and Shanghai Toujing Life Technology Co., Ltd., the intrabatch variation was kept below 10%, and the interbatch variation was controlled, stayed below 15% [10].

### 2.6. Incidence of Adverse Reaction

The patient's adverse symptoms such as nausea, vomiting, hypotension, and bone marrow suppression were carefully recorded.

### 2.7. Statistical Analysis

The data were analyzed by SPSS 21.0 software (IBM SPSS Statistics, USA), and the data were presented as mean ± standard deviation (SD) and tested by two-tailed Student's *t*-test. *P* < 0.05 was considered significant.

## 3. Results

### 3.1. Comparison of Clinical Intervention Effects

In this study, patients were divided into treatment group A (42 cases) and treatment group B (44 cases). No significant differences in general information were observed in patients between two groups (Table 1). As shown in Table 2, the total intervention effective rate of patients in treatment group B after this afatinib targeted therapy intervention was 84.09% (37/44), which was more significant than that of patients in treatment group A, which was 57.14% (24/42). The clinical intervention effect was more meaningful (*χ*^2^ = 7.678, *P* < 0.05).

### 3.2. Comparison of Immune Function

As shown in Table 3, before intervention, CD3^+^ (66.06 ± 8.24), CD4^+^ (27.64 ± 4.17), CD8^+^ (39.06 ± 5.04), and CD4^+^/CD8^+^ (0.64 ± 0.13) of patients in treatment group B were compared with those in treatment group A (65.05 ± 7.35, 27.07 ± 4.25, 39.05 ± 4.67, and 0.66 ± 0.14); there was no significant difference between the two (*F* = 5.06, 4.57, 4.33, 5.02, *P* > 0.01); after the intervention, the CD3^+^ (91.15 ± 12.53), CD4^+^ (50.24 ± 10.62), CD8^+^ (23.44 ± 4.31), and CD4^+^/CD8^+^ (1.86 ± 0.26) of patients in treatment group B were compared to those in treatment group A (77.07 ± 11.15, 35.17 ± 6.34, 32.61 ± 5.18, and 1.27 ± 0.14, respectively); the difference was significant (*t* = 4.14, 6.22, 6.76, 5.21, *P* < 0.01).

### 3.3. Comparison of Serum EGFR and pro-GRP Levels

Before intervention, there was no significant difference in serum EGFR levels (62.08 ± 11.67 and 61.21 ± 11.83) in treatment groups A and B (*t* = 0.164, *P* > 0.01); after intervention, serum EGFR levels in treatment groups A and B (58.34 ± 8.41 and 40.41 ± 6.26) were significantly decreased, and the serum EGFR level of patients in treatment group B (40.41 ± 6.26) was significantly lower than that of patients in treatment group A (58.34 ± 8.41), and there was a significant difference between the two (*t* = 13.683, *P* < 0.01). Before intervention, there was no significant difference in serum pro-GRP levels (265.38 ± 14.67 and 267.34 ± 14.71) in treatment groups A and B (*t* = 0.058, *P* > 0.01). The levels of pro-GRP (138.43 ± 19.56 and 106.41 ± 17.26) were significantly decreased, and the serum pro-GRP level (106.41 ± 17.26) of the patients in treatment group B was significantly lower than that of the patients in treatment group A (138.43 ± 19.56); there was a significant difference between the two (*t* = 20.163, *P* < 0.01, Table 4).

### 3.4. Adverse Reaction Comparison

As shown in Table 5, the total incidence of adverse reactions in treatment group B was 9.09% (4/44), which was significantly lower than that in treatment group A, 28.57% (12/42) (*P* < 0.001).

## 4. Discussion

In recent years, a large number of studies have found that the progression of malignant tumors is closely related to the immunocompromised patients [11, 12]. The antitumor immune function of patients can regulate the internal immune level of the body through different subsets of T cells, and CD4+ and CD8+ cellular immune factors are both important indicators of immune function, which can effectively reflect the level of immune function changes in the patient's body [13, 14]. This study found that the total intervention effective rate of patients in treatment group B after this afatinib targeted therapy intervention was 84.09% (37/44), which was more significant compared with 57.14% (24/42) in patients in treatment group A, indicating that the two groups' clinical intervention effect of the patients is more meaningful; at the same time, before the intervention, there was no significant difference in cellular immune factors such as CD4+ and CD8+ between the two groups of patients. The overall incidence of adverse reactions was 9.09%, which was significantly lower than that of treatment group A, which was 28.57%. This shows that afatinib targeted therapy can effectively improve the immune function of patients and reduce its adverse reactions. The reason for the analysis is that afatinib targeted therapy is an important molecule acting on the signaling pathway, which can effectively hinder the expansion of malignant tumor lesions and improve the patient's immune response ability, thereby improving their immune function and reducing adverse reactions [15, 16].

Tokito and other scholars have shown that serum tumor markers play a key role in the prognosis of patients with malignant tumors. Among them, EGFR and pro-GRP are widely used in the diagnosis and evaluation of non-small-cell lung cancer. Therefore, these two indicators are both important indicators for the evaluation of the disease in this study [17, 18]. Through the results of this study, it was found that before the intervention, there was no significant difference in the serum EGFR and pro-GRP levels of the patients in treatment groups A and B; after the intervention, the serum EGFR and pro-GRP levels in treatment groups A and B were significantly decreased, and the serum EGFR and pro-GRP levels of patients in treatment group B were significantly lower than those of patients in treatment group A, and there was a significant difference between the two. This indicates that afatinib targeted therapy can effectively improve serum EGFR and pro-GRP levels in patients. The reason is that afatinib targeted therapy can effectively inhibit EGFR-HER2 signaling and hinder the spread of tumor cells and is of great value to the anticancer effect and coagulation function of patients [19, 20].

In conclusion, the clinical intervention effect of afatinib targeted therapy in patients with non-small-cell lung cancer is significant, which not only helps to improve the patient's immune function but also effectively improves the patient's serum EGFR and pro-GRP levels and reduces the occurrence of adverse reactions, which can be widely used in clinical practice. However, the sample size of the current study is limited. Further study with large sample sized is needed in the future to further validate the effect of afatinib targeted therapy.

## Figures and Tables

**Table 1:** Comparison of the general information of the two groups of included patients.

Group	Case	Gender (male/female)	Age (year)	BMI (kg/m^2^)	ASA grade	
					Adenocarcinoma	Adenosquamous carcinoma
Treatment group A	42	24/18	56.87 ± 6.43	26.57 ± 5.32	20	22
Treatment group B	44	26/18	57.23 ± 6.33	26.74 ± 5.44	23	21
*χ*2/*t*		0.146	0.265	0.365	0.437	
*P*		*0.796*	0.645	0.568	0.567	

**Table 2:** Comparison of the clinical intervention effects of the two groups after the intervention of the included patients.

Group	Basically improved	Partial improvement	Stable condition	Disease progression	Total intervention effectiveness
Treatment group A (*n* = 42)	13 (30.95)	11 (26.19)	10 (23.81)	8 (19.05)	24 (57.14)
Treatment group B (*n* = 44)	24 (54.54)	13 (29.54)	5 (11.36)	2 (4.54)	37 (84.09)
*χ* ^2^	—				7.678
*P*	—				<0.05

**Table 3:** Comparing the immune function indexes of the two groups of patients before and after intervention.

Group	Treatment group A (*n* = 42)	Treatment group B (*n* = 44)	*F*_*t*_/*F*_*g*_	*P*_*t*_/*P*_*g*_
CD_3_^+^				
Before intervention	65.05 ± 7.35	66.06 ± 8.24	5.06/4.14	0.02/0.01
After intervention	77.07 ± 11.15^	91.15 ± 12.53^^∗^		
CD_4_^+^				
Before intervention	27.07 ± 4.25	27.64 ± 4.17	4.57/6.22	0.03/0.01
After intervention	35.17 ± 6.34^	50.24 ± 10.62^^∗^		
CD_8_^+^				
Before intervention	39.05 ± 4.67	39.06 ± 5.04	4.33/6.76	0.02/0.01
After intervention	32.61 ± 5.18^	23.44 ± 4.31^^∗^		
CD_4_^+^/CD_8_^+^				
Before intervention	0.66 ± 0.14	0.64 ± 0.13	5.02/5.21	0.02/0.01
After intervention	1.27 ± 0.14^	1.86 ± 0.26^^∗^		

Note: *F*_*t*_ and *P*_*t*_ are time statistics; *F*_*g*_ and *P*_*g*_ are between-group factor statistics; compared with treatment group A, ^∗^*P* < 0.01; compared with before intervention, ^*P* < 0.01.

**Table 4:** Comparison of serum EGFR and pro-GRP levels in the two groups of patients before and after intervention.

Group	Treatment group A (*n* = 42)	Treatment group B (*n* = 44)	*t*	*P*
Serum EGFR level				
Before intervention	62.08 ± 11.67	61.21 ± 11.83	0.164	>0.01
After intervention	58.34 ± 8.41	40.41 ± 6.26	13.683	<0.01
Serum pro-GRP level				
Before intervention	265.38 ± 14.67	267.34 ± 14.71	0.058	>0.01
After intervention	138.43 ± 19.56	106.41 ± 17.26	20.163	<0.01

**Table 5:** Adverse reactions after the intervention of the included patients in the two groups.

Group	Feel sick and vomit	Low blood pressure	Myelosuppression	Total occurrence rate
Treatment group A (*n* = 42)	4 (9.52)	2 (4.76)	4 (9.52)	12 (28.57)
Treatment group B (*n* = 44)	1 (2.27)	1 (2.27)	2 (4.54)	4 (9.09)
*χ* ^2^	—			7.764
*P*	—			0.001

## Data Availability

All data were within the manuscript.

## References

[1] Puliafito I., Esposito F., Raciti G. (2022). Metabolic complete tumor response in a patient withepidermal growth factor receptormutant non-small cell lung cancer treated with a reduced dose of afatinib. *Journal of International Medical Research*.

[2] Hadfield M. J., Turshudzhyan A., Shalaby K., Reddy A. (2022). Response with pembrolizumab in a patient with EGFR mutated non-small cell lung cancer harbouring insertion mutations in V834L and L858R. *Journal of Oncology Pharmacy Practice*.

[3] Metro G., Crinò L. (2011). The LUX-Lung clinical trial program of afatinib for non-small-cell lung cancer. *Expert Review of Anticancer Therapy*.

[4] Heigener D. F., Schumann C., Sebastian M. (2015). Afatinib in non-small cell lung cancer harboring uncommon EGFR mutations pretreated with reversible EGFR inhibitors. *The Oncologist*.

[5] Kim Y., Sun J., Park K. (2017). P3.01-023 first-line afatinib for non-small cell lung cancer in real world practice. *Journal of Thoracic Oncology*.

[6] Chung C. T., Yeh K. C., Lee C. H. (2020). Molecular profiling of afatinib-resistant non-small cell lung cancer cells in vivo derived from mice. *Pharmacological Research*.

[7] Miller V. A., Hirsh V., Cadranel J. (2012). Afatinib versus placebo for patients with advanced, metastatic non-small-cell lung cancer after failure of erlotinib, gefitinib, or both, and one or two lines of chemotherapy (LUX-Lung 1): a phase 2b/3 randomised trial. *The Lancet Oncology*.

[8] Liang W., Wu X., Fang W. (2014). Network meta-analysis of erlotinib, gefitinib, afatinib and icotinib in patients with advanced non-small-cell lung cancer harboring EGFR mutations. *PLoS One*.

[9] Hirsh V. (2011). Afatinib (BIBW 2992) development in non-small-cell lung cancer. *Future Oncology*.

[10] He J., Tan W., Tang X., Ma J. (2017). Variations in EGFR ctDNA correlates to the clinical efficacy of afatinib in non small cell lung cancer with acquired resistance. *Pathology & Oncology Research*.

[11] Harada T., Futamura S., Inoue Y., Sawada R., Okuda T., Kagawa K. (2019). P2.03-13 acquired resistance to afatinib in non-small cell lung cancer with EGFR G719X mutation. *Journal of Thoracic Oncology*.

[12] Isla D., De Castro J., Vidal O. J. (2015). Adverse events costs associated with erlotinib or afatinib in non-small cell lung cancer (Nsclc) patients with Egfr mutation-positive tumours. *Value in Health the Journal of the International Society for Pharmacoeconomics & Outcomes Research*.

[13] Yang Z., Hackshaw A., Feng Q. (2017). Comparison of gefitinib, erlotinib and afatinib in non-small cell lung cancer: a meta-analysis. *International Journal of Cancer*.

[14] Hochmair M., Holzer S., Burghuber O. C. (2016). Complete remissions in afatinib-treated non-small-cell lung cancer patients with symptomatic brain metastases. *Anti-Cancer Drugs*.

[15] Davies R. S., Brewster A. E., Button M. R., Lester J. F. (2016). 60 Afatinib in EGFR mutation positive advanced non-small cell lung cancer. *Lung Cancer*.

[16] Bowles D. W., Weickhardt A., Jimeno A. (2013). Afatinib for the treatment of patients with EGFR-positive non-small cell lung cancer. *Drugs of Today*.

[17] Tokito T., Ko R., Imamura C. (2019). P1.14-30 phase I study of afatinib plus bevacizumab in patients with advanced non-small cell lung cancer harboring EGFR mutations. *Journal of Thoracic Oncology*.

[18] Aredo J. V., Wakelee H. A., Neal J. W., Padda S. K. (2022). Afatinib After Progression on Osimertinib in EGFR -Mutated Non-Small Cell Lung Cancer. *Cancer Treatment and Research Communications*.

[19] Pakkala S., Ramalingam S. S. (2017). Epidermal growth factor receptor mutated advanced non-small cell lung cancer: a changing treatment paradigm. *Hematology/Oncology Clinics of North America*.

[20] Kato T., Yoshioka H., Okamoto I. (2015). Afatinib versus cisplatin plus pemetrexed in Japanese patients with advanced non-small cell lung cancer harboring activating EGFR mutations: subgroup analysis of LUX-lung 3. *Cancer Science*.

